# Modeling and simulation of railway safety management with public supervision and dynamic incentives: A four-party evolutionary game and system dynamics approach

**DOI:** 10.1371/journal.pone.0330100

**Published:** 2025-08-18

**Authors:** Xiaoxi Li, Xueru Cao, Jinyan Deng, Xinyuan Li

**Affiliations:** 1 Jiangxi Provincial Key Laboratory of Comprehensive Stereoscopic Traffic Information Perception and Fusion, East China Jiaotong University, Nanchang, China; 2 School of Transportation Engineering, East China Jiaotong University, Nanchang, China; 3 School of Tianyou, East China Jiaotong University, Nanchang, China; University of Hong Kong, HONG KONG

## Abstract

Railway accidents pose a significant threat to the industry, necessitating enhanced research into railway transportation safety. This study integrated a public oversight framework into the existing safety governance structure of railway transport operators, utilizing a four-party evolutionary game model and system dynamics for enhancement. Simulations conducted with Vensim software demonstrate that increased public supervision increases safety operation rates and improves the safety-related productivity of auxiliary enterprises. However, uncertainties in the evolutionary strategy process were identified. To address equilibrium fluctuations, a dynamic reward-punishment mechanism was developed. The optimized system achieved a safety operation rate of 99.7%, enhanced the safety-related productivity of the auxiliary enterprises to 93.2%, and increased the public supervision rate to 87.2%. These findings indicate that effective public participation and dynamic incentives can significantly improve safety management and prevent losses in railway sectors, offering valuable theoretical and practical insights for global railway enterprises.

## Background

### Evolution of the railroad safety management authorities

The implementation of safety management systems (SMS) in the railroad sector varies significantly across countries due to the influence of distinct regulatory bodies and operator-specific requirements [1–8]. Although no universal consensus exists on SMS components, essential elements are widely acknowledged. Despite this variation, the importance of SMS in the railway industry is well-recognized, leading to comprehensive adoption among operators. However, successful establishment and evolution of these systems require considerations beyond compliance. Substantial efforts have produced holistic SMS models, for example, the European Railway Agency established the “Safety Management System Wheel” to enhance safety management practices [9]. Similarly, the UK Office of Rail Regulation (ORR) developed the Railway Management Maturity Model for evaluating railway SMS effectiveness.

In China, railway safety standards are established through legislation enacted by multi-level regulatory bodies. These provisions primarily address accident management, safety oversight, and operational governance [5,10]. By the end of 2023, China’s railway network spanned 159,000 kilometers, transporting approximately 4.05 billion passengers and 4.98 billion tons of cargo (State Railway Administration, 2023). This extensive infrastructure necessitates a robust regulatory framework to ensure operational safety.

Significant regulatory changes followed the 2013 restructuring of railway utilities. Prior to this reform, China’s railway safety oversight underwent substantial administrative changes. The pivotal 2013 reorganization terminated the Ministry of Railways (MOR), transferring its critical infrastructure planning and policy mandates to the Ministry of Transport (MOT) [11]. Subsequently, the State Railway Administration (SRA) was established under the MOT, inheriting the MOR’s core administrative functions, while the China Railway Corporation (CRC) assumed commercial operations [11].

Pre-reform regulation operated through a four-tier hierarchy: the MOR’s central Department of Safety Regulation, 18 regional Railway Bureaus, Depot-level sections, and 845 station-level offices enabling nationwide real-time monitoring. Post-reform, this was streamlined to a two-level model: the SRA oversees seven Regional Railway Administrations (RRAs) (General Office of the State Council, 2013), which supervise 18 Railway Subsidiary Corporations (RSCs) under the CRC. This architecture delineates distinct regulatory roles, with the SRA governing both the CRC and all RSCs, while RRAs enforce compliance within their jurisdictions-transitioning from centralized bureaucratic control to integrated governmental-corporate oversight.

### Safety management of China Railway Nanchang Bureau Group Co., Ltd

#### Organizational structure and safety oversight.

**Fig 1:**
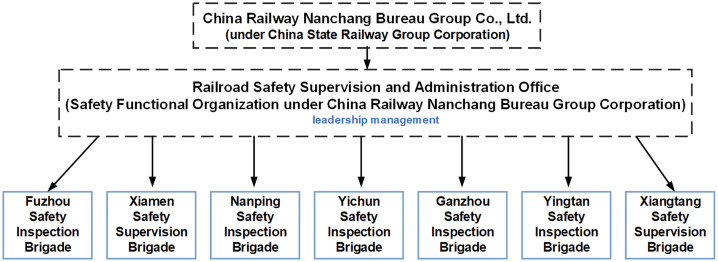
The current organizational structure of safety management department of China Railway Nanchang Bureau Group Co., Ltd.

China Railway Nanchang Bureau Group Company Limited (China Railway Nanchang Bureau Group Co., Ltd.), established in 1996 and restructured in 2004 following a merger with Fuzhou Railway Branch, was formally listed in November 2017. As a critical national railway hub, Nanchang Railway connects the Yangtze River Delta and the Pearl River Delta regions while facilitating east-west and north-south transportation corridors. It further serves as a comprehensive transportation hub in Jiangxi Province with significant national influence. Given these strategic roles, China Railway Nanchang Bureau Group Co., Ltd. was selected as the research case study. The current organizational structure of the safety management department within the China Railway Nanchang Bureau Group Co., Ltd. is illustrated in Fig 1. This illustrates the internal safety management organization of the China Railway Nanchang Bureau Group Co., Ltd. [12]. Meanwhile, regulatory oversight is conducted by the Shanghai Railway Supervision Administration (SRSA) – a local entity under the SRA responsible for supervising the daily operations of the China Railway Nanchang Bureau Group Co., Ltd.

#### Accident data statistics and analysis.

Based on administrative penalty announcements from the SRSA and circulars regarding supervised rail equipment at licensed enterprises, Tables 1 and 2 summarize safety incident frequencies involving China Railway Nanchang Bureau Group Co., Ltd. and local auxiliary enterprises since 2017. The penalties of China Railway Nanchang Bureau Group Co., Ltd. comprise violations of production safety regulations, while auxiliary enterprise penalties reflect SRSA sanctions for inadequate safety measure implementation.

**Table 1:** Safety Operation Records of China Railway Nanchang Bureau Group Co., Ltd.

Year	Number of administrative penalties	Number of supervisory and inspection briefings for railroad special equipment administratively licensed enterprises	Replenishment
2023	3	1	2 interviews
2022	4	0	not have
2021	3	1	not have
2020	3	1	not have
2019	4	0	not have
2018	2	1	not have
2017	1	0	not have

**Table 2:** Record of safety production in auxiliary enterprises.

Year	Number of administrative penalties	Number of supervisory and inspection briefings for railroad special equipment administratively licensed enterprises
2023	13	1
2022	12	0
2021	14	2
2020	13	1
2019	14	1
2018	12	0
2017	9	3

Table 3 documents a record of public complaints and commendations related to the supervision of China Railway Nanchang Bureau Group Co., Ltd. for each quarter since 2022.

**Table 3:** Public Oversight (Rail Transportation).

Quarter	Number of letters in the complaints category	Number of letters of commendation
First quarter of 2024	9	0
Fourth quarter of 2023	7	0
Third quarter of 2023	16	1
Second quarter of 2023	7	0
First quarter of 2023	12	0
Fourth quarter of 2022	19	1
Third quarter of 2022	16	0
Second quarter of 2022	15	0
First quarter of 2022	15	0
Fourth quarter of 2021	9	1
Third quarter of 2021	23	0
Second quarter of 2021	26	0
First quarter of 2021	19	0
First quarter of 2020	6	1

China’s recent railway institutional reforms established a multi-stakeholder safety accountability framework integrating enterprise accountability, workforce participation, governmental oversight, industry self-regulation, and societal monitoring – transitioning from pyramidal hierarchies to decentralized governance [11]. While comparative analyses indicate this flattened architecture enhances regulatory efficiency [13], its implementation diverges from conventional flat management principles and retains structural parallels with regulatory regimes. Crucially, eliminating safety supervision roles at depot and station levels has eroded grassroots regulatory mechanisms, substantially reducing regulatory staffing. This transformation shifted regulator-regulated relationships from hierarchical subordination to inter-organizational coordination, intensifying information asymmetry risks inherent in externalized oversight. Although reforms dismantled institutional protectionism, resource constraints concurrently diminished regulators’ real-time monitoring capacity, creating paradoxical tensions between bureaucratic independence and operational efficacy.

### Literature review and research focus

Railroad safety is systemically influenced by stakeholder behaviors. Research indicates accidents often originate from failures across the entire safety management system [14]. Consequently, effective accident prevention requires both robust systemic frameworks and understanding behavioral interactions within them. Key concepts addressing this dynamic are summarized in the following literature review.

### Safety management system

The high frequency of railroad accidents necessitates continuous safety management improvements within the industry. Concurrently, safety management system (SMS) have evolved into defined frameworks of activities fulfilling safety responsibilities [15–18]. This conceptual progression stems from multidisciplinary theoretical advances that fundamentally reshape safety governance. Since early sequential causality models, core accident prevention paradigms, including energy transfer theory, systemic failure models, and organizational drift constructs, have undergone iterative refinement through empirical validation and cross-domain application [5,19]. Such theoretical development continuously informs adaptive risk mitigation architectures in industrial SMS [16,20]. Technological integration and interdisciplinary synthesis have driven increasing system complexity, transforming safety models into interconnected holistic frameworks [3,4,21]. Two predominant model types emerge: accident models and organizational models [22–24]. Contemporary safety science features significant theoretical syntheses through multidisciplinary modeling. Building upon Reason’s seminal analysis of complex systems [25], researchers formulated a causation-control framework that integrates human factor dynamics and systemic feedback mechanisms. Concurrently, the Bowtie-Bayesian synthesis model has emerged as a robust methodological innovation for probabilistic risk assessment in socio-technical systems [16]. This theoretical progression underpins the tripartite evolution of safety governance frameworks across [16]: conceptual development, formalization of accident causation theories; operational implementation, design of adaptive risk control architectures; regulatory standardization, institutionalization of safety management protocols. The transition from basic safety equipment to comprehensive systematic management underscores accident prevention’s critical role in modern enterprise safety.

### Evolutionary game theory

Game theory emerged in the mid-20th century as a formal framework for strategic interactions [21,26,27]. Early models assumed perfectly rational homo economicus agents [11]. By the 1980s, evolutionary game theory had developed into a mature and widely accepted modeling framework. Evolutionary game theory uses replicator dynamics to describe the learning and evolutionary mechanisms of individuals in the process of railroad safety regulation. Replicator dynamics gives a dynamic description of the trend of behavior change of a finite rational group in a system [28]. It is the basic model of evolutionary game theory, reflecting the selection effect of the system. The concept of replicator dynamics posits that individuals who choose unfavorable strategies tend to imitate those who choose favorable strategies. This approach causes the proportion of individuals choosing favorable strategies to increase, and the system eventually reaches a stable state. These dynamics formalize population-level behavioral evolution through differential equation systems [28–30], capturing emergent trends in the selection of group strategies. The framework formalizes how boundedly rational populations shift toward advantageous strategies: agents adopting suboptimal strategies progressively emulate higher-payoff counterparts through social learning, increasing the prevalence of dominant behaviors until evolutionarily stable equilibria emerge. This selection-driven convergence correlates strategy growth rates with relative payoff advantages. Hausken’s generalized formulations [31] extend this to intergroup strategy migration via discrete/continuous-time frameworks, bridging micro-level imitation patterns with macro-level institutional stability in governance contexts [11].

### Rent-seeking

Management structures, industry policies, and close relationships between firms and government regulators can lead to rent-seeking behavior. The concept of rent-seeking theory was first introduced in the mid-1960s. First formalized by Krueger [32], as a consequence of government market intervention, rent-seeking constitutes direct unproductive activities such as bribery, corruption, and power abuse [33]. These behaviors increase transaction costs, distort resource allocation, and precipitate market failure, necessitating effective controls to maintain economic order. Rent-seeking behavior is typically defined as the definition, redistribution, modification, or weakening of property rights. Benson [34] believes that rent-seeking behavior is the redistribution of existing property by individuals or groups. Rent seeking behavior implies taking uncompensated value from others without contributing anything to production. Rent-seeking behavior can take many forms, such as firms spending money to lobby governments for subsidies and firms colluding with regulators [35]. In the railway industry, the lack of standardized third-party certification will lead to non-market competition models, which will further undermine operational integrity and the effectiveness of safety governance.

While prior research extensively examines SMS concepts, systemic dynamics in railway contexts remain underexplored [36–38]. Consequently, developing proactive safety governance frameworks and analyzing systemic risk interdependencies are critical for reducing railway accident incidence [39]. This study addresses this gap through a case study of China Railway Nanchang Bureau Group Co., Ltd. This study introduces a public supervision mechanism into SMS, establishing a four-party evolutionary game model comprising regulatory bodies, supervisory agents, and supervisees. Specifically, SRSA serves as the governmental regulator, directly overseeing operational safety at China Railway Nanchang Bureau Group Co., Ltd. and regional safety-supportive enterprises; simultaneously, SRSA leverages public participation to supplement supervisory capacity by encouraging reports of safety violations at China Railway Nanchang Bureau Group Ltd. and affiliated enterprises. This optimized SMS integrates evolutionary game theory with system dynamics simulations to quantitatively assess risk mitigation efficacy and regulatory effectiveness.

### Case study and insights

#### Construction of the optimized safety management system.

**Fig 2:**
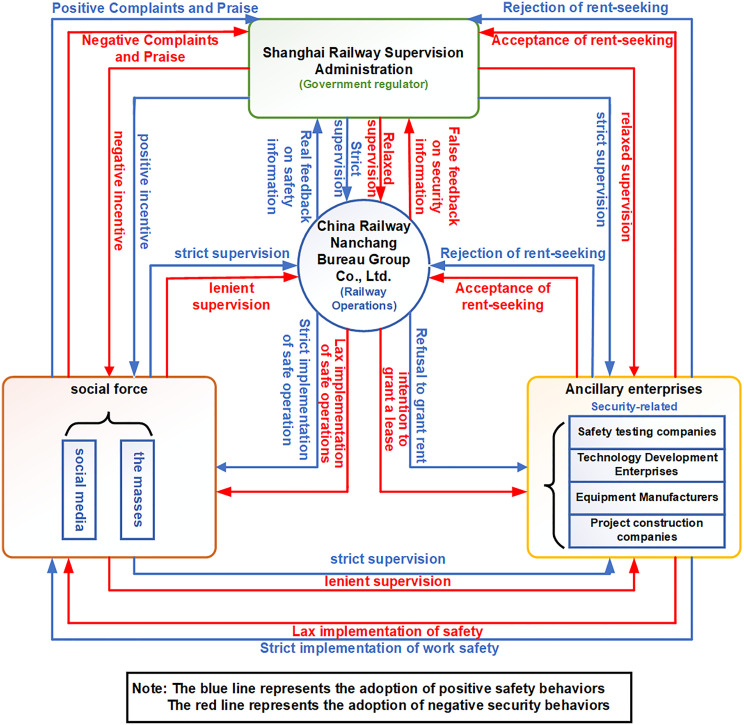
Optimized safety management system of China Railway Nanchang Bureau Group Co., Ltd. (Also: logic relationship diagram of the four-party evolutionary game model).

During the regulatory process, the SRSA may adopt proactive or passive oversight over China Railway Nanchang Group Co., Ltd. and local safety-related auxiliary enterprises. Proactive supervision represents maximal intervention (e.g., real-time monitoring), while passive supervision denotes minimal engagement (e.g., no oversight). China Railway Nanchang Group Co., Ltd. and auxiliary enterprises face strategic choices: implement rigorous safety protocols for standard profits or engage in unsafe practices for maximized gains. Concurrently, the public may report safety violations or remain passive. This complex interaction among SRSA, China Railway Nanchang Bureau Group Co., Ltd., auxiliary safety enterprises, and the public necessitates regulatory coordination through legal enhancements to ensure railway safety. Fig 2 illustrates both the optimized safety management system and the four-party evolutionary game’s logical structure, comprising SRSA, China Railway Nanchang Bureau Group Co., Ltd., auxiliary safety enterprises, and the public.

### Construction of the evolutionary game model

#### Four-party role descriptions.

Chinese government directs the national railway safety management system through designated regulatory officials. As shown in Fig 2, the SRSA, which is subordinate to the National Railway Administration, serves as the primary regulatory authority.

China Railway Nanchang Bureau Group Co., Ltd., the principal operator under SRSA supervision, formulates safety objectives and manages operations including dispatch, command, monitoring, early warning, risk management, and other tasks.

Social forces function as safety stakeholders, providing demand signals, guidance, and supervision. Some railway sections under the jurisdiction of China Railway Nanchang Bureau Group Co., Ltd. traverse complex terrain with significant elevation variations, resulting in natural disaster risks, supervision challenges, and continuous monitoring limitations that necessitate comprehensive public participation.

Auxiliary enterprises deliver essential safety technologies and services. Strategic collaboration among manufacturing, safety service, and high-tech enterprises is critical for advancing railway infrastructure resilience. China Railway Nanchang Bureau Group Co., Ltd. enhances disaster preparedness through technological partnerships with these entities.

Due to the complexity of the four parties involved and the difficulty of controlling variables in real-world experiments, and given that a rigorous simulation can provide a basis for constructing of an optimized railway safety management system, this study primarily employs simulation methods for research.

#### Stating assumptions.

The evolutionary game framework operates under four core assumptions:

Assumption 1: All participants are assumed to possess finite rationality. Under finite rationality, the optimal strategy does not result from a one-time choice but gradually evolves from the participants’ long-term decision-making and learning, ultimately achieving an equilibrium that may be affected by various variables.

Assumption 2: Unsafe production practices are operationally defined as non-criminal infractions under current Chinese regulatory statutes governing industrial safety.

Assumption 3: All organizational actors possess a comprehensive understanding of statutory safety liability frameworks and associated legal consequences.

Assumption 4: The rational economic calculus of railway operators (e.g., China Railway Nanchang Bureau Group Co., Ltd.) prioritizes risk-adjusted returns, where marginal revenues from safety regulation noncompliance systematically exceed projected accident-induced liabilities, which is a necessary precondition for suboptimal safety investment decisions.

Friedman’s analytical framework [40] assesses evolutionary equilibrium stability through Jacobian matrix eigenvalue decomposition. However, this method only identifies terminal evolutionary stable states (ESS), failing to characterize transient dynamics or quantify endogenous regulatory factors in railway safety governance.

Evolutionary processes inherently feature intra-group behavioral feedback, where agents iteratively emulate superior strategies through iterative payoff comparisons and observational learning [11]. To address the temporal dimension of strategic adaptation, system dynamics methodologies provide robust computational tools for modeling both equilibrium convergence trajectories and solution basin stability thresholds, effectively capturing the nonlinear interdependencies between institutional constraints and agent learning behaviors [11,41–43].

### Model parameter settings

The parameters involved in the model are listed in Table 4.

**Table 4:** Parameter assumptions involved in the model.

Participant	Norm	Significance of the indicators	Indicator value range
Shanghai Railway Supervision Administration	*x*	Rate of implementation of safety supervision	0 ≤ *x* ≤ 1
	*C*_*S*_	Supervision of Nanchang Bureau and business inputs	*C*_*S*_ ≥ 0
	*C*_*SP*_	Handling of public complaint inputs	*C*_*SP*_ ≥ 0
	*L*_*SN*_	Losses due to inadequate supervision of Nanchang Bureau	*L*_*SN*_ ≥ 0
	*L*_*SB*_	Losses from inadequate supervision of local businesses	*L*_*SB*_ ≥ 0
	*P*_*SN*_	Fines for poor safety operations at Nanchang Bureau	*P*_*SN*_ ≥ 0
	*P*_*SB*_	Fines for poor safety at local businesses	*P*_*SB*_ ≥ 0
	*R*_*SN*_	Strictly safe operation incentives for Nanchang Bureau	*R*_*SN*_ ≥ 0
	*R*_*SB*_	Strict safety incentives for local businesses	*R*_*SB*_ ≥ 0
	*R*_*SPN*_	Rewards for public scrutiny of Nanchang Bureau	*R*_*SPN*_ ≥ 0
	*R*_*SPB*_	Rewards for public scrutiny of local businesses	*R*_*SPB*_ ≥ 0
China Railway Nanchang Bureau Group Co., Ltd.	*y*	Safe Operation Implementation Rate	0 ≤ *y* ≤ 1
	*NC*_*N*_	Revenue from day-to-day implementation of security operations	*NC*_*N*_ ≥ 0
	*NC*_*E*_	Operating additional revenue against security	*NC*_*E*_ ≥ 0
	*L*_*BN*_	Unsafe production by local businesses takes its toll	*L*_*BN*_ ≥ 0
Local Ancillary Enterprises	*z*	Productive safety implementation rate	0 ≤ *z* ≤ 1
	*B*_*N*_	Daily implementation of security production revenue	*B*_*N*_ ≥ 0
	*B*_*E*_	Additional income from breach of security	*B*_*E*_ ≥ 0
The Masses	*w*	Participation rate in social monitoring	0 ≤ *w* ≤ 1
	*C*_*P*_	Supervisory expenditures on Nanchang Bureau and local enterprises	*C*_*P*_ ≥ 0
	*L*_*NP*_	Neglect of unsafe operating losses at Nanchang Bureau	*L*_*NP*_ ≥ 0
	*L*_*BP*_	Neglect of unsafe production losses in local businesses	*L*_*BP*_ ≥ 0

Table 5 presents a four-player game matrix that contains 16 strategic permutations derived from binary choice sets assigned to each participant. Within this framework:

**Table 5:** Quadratic strategy game set.

Gaming Strategy				Shanghai Railway Supervision Administration			
				$\alpha_{1}(x)$		$\alpha_{2}(1-x)$	
				China Railway Nanchang Bureau Group Co., Ltd.			
				$\beta_{1}(y)$	$\beta_{2}(1-y)$	$\beta_{1}(y)$	$\beta_{2}(1-y)$
Local Enterprise	$\gamma_{1}(z)$	Public	$\theta_{1}(w)$	$\left(\alpha_{1},\beta_{1},\gamma_{1},\theta_{1}\right)$	$\left(\alpha_{1},\beta_{2},\gamma_{1},\theta_{1}\right)$	$\left(\alpha_{2},\beta_{1},\gamma_{1},\theta_{1}\right)$	$\left(\alpha_{2},\beta_{2},\gamma_{1},\theta_{1}\right)$
			$\theta_{2}(1-w)$	$\left(\alpha_{1},\beta_{1},\gamma_{1},\theta_{2}\right)$	$\left(\alpha_{1},\beta_{2},\gamma_{1},\theta 2\right)$	$\left(\alpha_{2},\beta_{1},\gamma_{1},\theta_{2}\right)$	$\left(\alpha_{2},\beta_{2},\gamma_{1},\theta_{2}\right)$
	$\gamma_{2}(1-z)$	Public	$\theta_{1}(w)$	$\left(\alpha_{1},\beta_{1},\gamma_{2},\theta_{1}\right)$	$\left(\alpha_{1},\beta_{2},\gamma_{2},\theta_{1}\right)$	$\left(\alpha_{2},\beta_{1},\gamma_{1},\theta_{1}\right)$	($\alpha_{2},\beta_{2},\gamma_{2},\theta_{1})$
			$\theta_{2}(1-w)$	$\left(\alpha_{1},\beta_{1},\gamma_{2},\theta_{2}\right)$	$\left(\alpha_{1},\beta_{2},\gamma_{2},\theta_{2}\right)$	$\left(\alpha_{2},\beta_{1},\gamma_{1},\theta_{2}\right)$	$\left(\alpha_{2},\beta_{2},\gamma_{2},\theta_{2}\right)$

(1) Regulatory fiscal flows

Penalties imposed by the SRSA create institutional revenue, while simultaneously generating operational liabilities for China Railway Nanchang Bureau Group Co., Ltd. and regional safety-critical enterprises. Conversely, incentive payments constitute budgetary outlays for the SRSA but provide income for the aforementioned enterprises and public beneficiaries.

(2) Enforcement conditionality

Rewards and penalties are triggered only when monitoring is exercised by either the SRSA or civil oversight coalitions.

Under the stated hypothesis, if the SRSA, China Railway Nanchang Bureau Group Co., Ltd., local safety auxiliary enterprises and the public each select their first strategy, the resulting profile is (*α*_1_,*β*_1_,*γ*_1_,*θ*_1_). The detailed procedure codes of payoff matrix calculation are available in S1 File. Tables 6 and 7 report the corresponding expected payoffs for these four agents.

**Table 6:** Matrix of benefits to the Shanghai Railway Supervision Administration and the public.

Payoff matrix	Shanghai Railway Supervision Administration	Public
$\left(\alpha_{1},\beta_{1},\gamma_{1},\theta_{1}\right)$	$-C_{S}-C_{SP}-R_{SN}-R_{SB}$	$-C_{SP}$
$\left(\alpha_{1},\beta_{2},\gamma_{1},\theta_{1}\right)$	$-C_{S}-C_{SP}+P_{SN}-R_{SB}-R_{SPN}$	$-C_{SP}+\;R_{SPN}$
$\left(\alpha_{2},\beta_{1},\gamma_{1},\theta_{1}\right)$	$-C_{SP}-R_{SN}-R_{SB}$	$-C_{SP}$
$\left(\alpha_{2},\beta_{2},\gamma_{1},\theta_{1}\right)$	$-C_{SP}+P_{SN}-R_{SB}-R_{SPN}$	$-C_{SP}+{\;R}_{SPN}$
$\left(\alpha_{1},\beta_{1},\gamma_{1},\theta_{2}\right)$	$-C_{S}-R_{SN}-R_{SB}$	0
$\left(\alpha_{1},\beta_{2},\gamma_{1},\theta_{2}\right)$	$-C_{S}\;+P_{SN}-R_{SB}$	0
$\left(\alpha_{2},\beta_{1},\gamma_{1},\theta_{2}\right)$	0	0
$\left(\alpha_{2},\beta_{2},\gamma_{1},\theta_{2}\right)$	$-L_{SN}$	$-L_{NP}$
$\left(\alpha_{1},\beta_{1},\gamma_{2},\theta_{1}\right)$	$-C_{S}-C_{SP}+P_{SB}-R_{SN}-R_{SPB}$	$-C_{SP}+\;R_{SPB}$
$\left(\alpha_{1},\beta_{2},\gamma_{2},\theta_{1}\right)$	$-C_{S}-C_{SP}+P_{SB}+P_{SN}-R_{SPN}-R_{SPB}$	$-C_{SP}+\;R_{SPN}+\;R_{SPB}$
$\left(\alpha_{2},\beta_{1},\gamma_{2},\theta_{1}\right)$	$-C_{SP}+P_{SB}\;-R_{SN}-R_{SPB}$	$-C_{SP}\;+{\;R}_{SPB}$
$\left(\alpha_{2},\beta_{2},\gamma_{2},\theta_{1}\right)$	$-C_{SP}+P_{SB}+P_{SN}-R_{SPN}-R_{SPB}$	$-C_{SP}+\;R_{SPN}+\;R_{SPB}$
$\left(\alpha_{1},\beta_{1},\gamma_{2},\theta_{2}\right)$	$-C_{S}\;+P_{SB}\;-R_{SN}$	0
$\left(\alpha_{1},\beta_{2},\gamma_{2},\theta_{2}\right)$	$-C_{S}\;+P_{SB}+P_{SN}$	0
$\left(\alpha_{2},\beta_{1},\gamma_{2},\theta_{2}\right)$	$-L_{SB}$	$-L_{BP}$
$\left(\alpha_{2},\beta_{2},\gamma_{2},\theta_{2}\right)$	$-L_{SN}-L_{SB}$	$-L_{NP}-L_{BP}$

**Table 7:** Payoff matrix of China Railway Nanchang Bureau Group Co., Ltd. and safety ancillary enterprises.

Payoff matrix	China Railway Nanchang Bureau Group Co., Ltd.	Security Supporting Enterprises
$\left(\alpha_{1},\beta_{1},\gamma_{1},\theta_{1}\right)$	${NC}_{N}+R_{SN}$	$B_{N}+R_{SB}$
$\left(\alpha_{1},\beta_{2},\gamma_{1},\theta_{1}\right)$	${NC}_{N}+{NC}_{E}-P_{SN}$	$B_{N}+R_{SB}$
$\left(\alpha_{2},\beta_{1},\gamma_{1},\theta_{1}\right)$	${NC}_{N}+R_{SN}$	$B_{N}+R_{SB}$
$\left(\alpha_{2},\beta_{2},\gamma_{1},\theta_{1}\right)$	${NC}_{N}+{NC}_{E}-P_{SN}$	$B_{N}+R_{SB}$
$\left(\alpha_{1},\beta_{1},\gamma_{1},\theta_{2}\right)$	${NC}_{N}+R_{SN}$	$B_{N}+R_{SB}$
$\left(\alpha_{1},\beta_{2},\gamma_{1},\theta_{2}\right)$	${NC}_{N}+{NC}_{E}-P_{SN}$	$B_{N}+R_{SB}$
$\left(\alpha_{2},\beta_{1},\gamma_{1},\theta_{2}\right)$	${NC}_{N}$	$B_{N}$
$\left(\alpha_{2},\beta_{2},\gamma_{1},\theta_{2}\right)$	${NC}_{N}+{NC}_{E}$	$B_{N}$
$\left(\alpha_{1},\beta_{1},\gamma_{2},\theta_{1}\right)$	${NC}_{N}+R_{SN}$	$B_{N}+B_{E}-P_{SB}$
$\left(\alpha_{1},\beta_{2},\gamma_{2},\theta_{1}\right)$	${NC}_{N}+{NC}_{E}-P_{SN}$	$B_{N}+B_{E}-P_{SB}$
$\left(\alpha_{2},\beta_{1},\gamma_{2},\theta_{1}\right)$	${NC}_{N}+R_{SN}$	$B_{N}+B_{E}-P_{SB}$
$\left(\alpha_{2},\beta_{2},\gamma_{2},\theta_{1}\right)$	${NC}_{N}+{NC}_{E}-P_{SN}$	$B_{N}+B_{E}-P_{SB}$
$\left(\alpha_{1},\beta_{1},\gamma_{2},\theta_{2}\right)$	${NC}_{N}+R_{SN}$	$B_{N}+B_{E}-P_{SB}$
$\left(\alpha_{1},\beta_{2},\gamma_{2},\theta_{2}\right)$	${NC}_{N}+{NC}_{E}-P_{SN}$	$B_{N}+B_{E}-P_{SB}$
$\left(\alpha_{2},\beta_{1},\gamma_{2},\theta_{2}\right)$	${NC}_{N}-L_{BN}$	$B_{N}+B_{E}$
$\left(\alpha_{2},\beta_{2},\gamma_{2},\theta_{2}\right)$	${NC}_{N}+{NC}_{E}-L_{BN}$	$B_{N}+B_{E}$

Let *S*_1_ denote the SRSA’s payoff under active supervision and *S*_2_ its payoff under non-supervision. When the SRSA oversees China Railway Nanchang Bureau Group Co., Ltd. and the safety-critical enterprises, *S*_1_ is the weighted product of the SRSA’s utility function and the joint probability distribution over the strategies of the three non-regulatory players. Solving the resulting system of simultaneous equations yields *S*_1_. This computational paradigm constructs simultaneous equations through strategic interaction probabilities, reflecting the benefit synthesis mechanism within multi-agent gaming environments. Following the same methodological framework, the payoff expression for *S*_2_ is derived via synergistic interactions of strategic variables (see Eqs (1) and (2) in S2 File Appendix A). Similarly, the structured formulation of expected returns for China Railway Nanchang Bureau Group Co., Ltd. (see Eqs (3) and (4) in S2 File Appendix A) is established on a multidimensional interaction model incorporating regulatory intensity, corporate compliance strategies, and public engagement levels. Here, penalty-reward parameters serve as core moderating variables, encoding the dynamic impacts of institutional incentives on strategic evolution. The expressions for the expected returns to the safety-supportive enterprises are given as Eqs (5) and (6) in S2 File Appendix A, and the expected returns to the public are given as Eqs (7) and (8) in S2 File Appendix A.

‾*S* is the individual earnings of the SRSA, which is supervised by the selection strategy. The expected average return of the SRSA is:


$$\bar{S}=xS_{1}+(1-x)S_{2}$$ (9)

The equation for the change in the proportion of SRSA accounted for by the regulatory strategy is:


$$dx/dt\;=\;x(S_{1}\;-\bar{S})$$ (10)

Then we substitute *S*_1_ into Eq (10). The temporal evolution of the mixed strategy variable *x*, corresponding to SRSA’s strategic adaptation dynamics, is mathematically governed by Eq (9). This differential formalism characterizes the time-dependent behavioral adjustments in the regulator’s policy portfolio under bounded rationality constraints. The value of *x* increases when *S*_1_ is greater than ‾*S*. The value of *x* falls when *S*_1_ is less than ‾*S*.

### Evolutionary stability analysis

Evolutionary game theory posits that equilibrium attainment occurs when strategic profiles exhibit invariance across all agents, signifying the ESS through iterative adaptation processes. This equilibrium condition reflects Nash equilibrium refinements under bounded rationality constraints, i.e., the rate of change of *x, y, z* and *w* are all 0. Therefore, combining Eqs (11) to (14) and making Eqs (11) to (14) equal to 0, results in the solution of the joint equations of the equations. This implies that the strategy adjustment rate diminishes to zero, driving the dynamical system towards a stationary state where evolutionary equilibrium is achieved, corresponding to the concept of an evolutionarily stable strategy.


$$dx/dt\;=\;x(1-x)(S_{1}\;–\;S_{2})\;=\;0$$ (11)

$$dy/dt\;=\;y(1-y)(NC_{1}\;–\;NC_{2})\;=\;0$$ (12)

$$dz/dt\;=\;z(1-z)(B_{1}\;–\;B_{2})\;=\;0$$ (13)

$$dw/dt\;=\;w(1-w)(P_{1}\;–\;P_{2})\;=\;0$$ (14)

Solving the system of nonlinear Eqs (11) to (14) yields an evolutionary game between the SRSA, Nanchang Bureau, local safety-supportive firms and the public. This consists of 16 pure strategy equilibria:


$$E_{1}=(0,0,0,0),\;E_{2}=(0,0,0,1),\;E_{3}=(0,0,1,1),\;E_{4}=(0,0,1,0)$$

$$E_{5}=(0,1,0,0),\;E_{6}=(0,1,0,1),\;E_{7}=(0,1,1,0),\;E_{8}=(0,1,1,1)$$

$$E_{9}=(1,0,0,0),\;E_{10}=(1,0,0,1),\;E_{11}=(1,0,1,1),\;E_{12}=(1,0,1,0)$$

$$E_{13}=(1,1,0,0),\;E_{14}=(1,1,0,1),\;E_{15}=(1,1,1,0),\;E_{16}=(1,1,1,1)$$

And the remaining 19 mixed-strategy equilibria: $E^{*}=(x^{*},\;y^{*},\;z^{*},\;w^{*})$.

Ritzberger and Weibull [44] proved that stable outcomes in multi-population evolutionary games coincide with strict Nash equilibria, which must be pure-strategy profiles. Leveraging this result, this work examines the stability characteristics of 16 pure-strategy equilibrium points that emerge in the four-party evolutionary game among the SRSA, China Railway Nanchang Bureau Group Co. Ltd., local safety auxiliary enterprises and the public. The replicator dynamics for these four populations are derived and the equilibrium points are computed. Since small perturbations in initial conditions or parameters can shift the equilibrium, each candidate must be tested for local stability. Only stable equilibria can be qualified as evolutionarily stable strategies. According to the replication dynamic equations of the participant, the Jacobi matrix of the four-party evolutionary game replication dynamic system can be obtained (see S2 File Appendix B for the Jacobi matrix). The detailed procedure codes of Jacobi matrix solution are available in S3 File.

Within the four-party evolutionary game system, the asymptotic stability characteristics of strategic interactions between SRSA, China Railway Nanchang Bureau Group Co., Ltd., local safety-supporting enterprises, and the public can be quantitatively determined through the Lyapunov’s first method [45]. This requires the construction of a Jacobi matrix for equilibrium points and subsequent eigenvalue analysis. The resulting stable equilibria are presented in Table 8.

**Table 8:** Fixed point analysis.

stabilization point	eigenvalue *λ*_*1*_*,λ*_*2*_*,λ*_*3*_*,λ*_*4*_	positive or negative	stability
(0,0,0,0)	$-B_{E}\;,\;-{NC}_{E},L_{SB}-C_{S}+L_{SN}+P_{SB}+P_{SN},$ $L_{BP}-C_{P}+L_{NP}+R_{SPN}+R_{SPB}$	(-,-,*,*)	precarious
(0,0,0,1)	$-C_{S},P_{SB}-B_{E}+R_{SB},P_{SN}-{NC}_{E}+R_{SN},$ $C_{P}-L_{BP}-L_{NP}-R_{SPN}-R_{SPB}$	(-,*,*,*)	precarious (Condition 2)
(0,0,1,0)	$-B_{E},-{NC}_{E},L_{NP}-C_{P}\;+R_{SPN},$ $L_{SN}-C_{S}+P_{SN}-\;R_{SB}$	(+,-,*,*)	precarious
(0,0,1,1)	$-C_{S},C_{P}-L_{NP}-R_{SPN},B_{E}-P_{SB}-R_{SB},$ $P_{SN}-{NC}_{E}+R_{SN}$	(-,*,*,*)	stabilise (Conditions 1*、2*)
(0,1,0,0)	${NC}_{E},\;-B_{E}\;,L_{BP}-C_{P}+R_{SPB},$ $L_{SB}-C_{S}+P_{SB}-R_{SN}$	(+,-,*,*)	precarious
(0,1,0,1)	$-C_{S},-L_{BP}+C_{P}-R_{SPB},{NC}_{E}-P_{SN}-R_{SN},$ $-B_{E}+P_{SB}+R_{SB}$	(-,*,*,*)	precarious (Conditions 1、2)
(0,1,1,0)	$B_{E},{NC}_{E},-C_{P},$ $-C_{S}-R_{SB}-R_{SN}$	(+, + ,-,-)	precarious
(0,1,1,1)	$C_{P},-C_{S},B_{E}-P_{SB}-R_{SB},$ $-P_{SN}+{NC}_{E}-R_{SN}$	(+,-,*,*)	precarious
(1,0,0,0)	$-B_{E}+P_{SB}+R_{SB}\;,\;-C_{P}+R_{SPB}+R_{SPN},\;P_{SN}-{NC}_{E}+R_{SN},$ $C_{S}\;-L_{SB}\;-L_{SN}\;-P_{SN}-\;P_{SB}$	(*,*,*,*)	precarious (Condition 2)
(1,0,0,1)	$C_{S},C_{P}-R_{SPB}-R_{SPN},-B_{E}+P_{SB}+R_{SB}\;,$ $P_{SN}-{NC}_{E}+R_{SN}$	(+,*,*,*)	precarious
(1,0,1,0)	$-C_{P}+R_{SPN},B_{E}-P_{SB}-R_{SB}\;,{\;P}_{SN}-{NC}_{E}+R_{SN},$ $C_{S}\;-L_{SN}\;-P_{SN}+\;R_{SB}$	(*,*,*,*)	precarious (Condition 3)
(1,0,1,1)	$C_{S},C_{P}-R_{SPN},B_{E}-P_{SB}-R_{SB}\;,$ $P_{SN}-{NC}_{E}+R_{SN}$	(+,*,*,*)	precarious
(1,1,0,0)	$-C_{P}+R_{SPB},\;-P_{SN}+{NC}_{E}-R_{SN},\;-B_{E}+P_{SB}+R_{SB}\;,$ $C_{S}\;-L_{SB}\;-P_{SB}+\;R_{SN}$	(*,*,*,*)	stabilise (Conditions 1、2)
(1,1,0,1)	$C_{S},C_{P}-R_{SPB},\;-P_{SN}+N_{CE}-R_{SN},$ $-B_{E}+P_{SB}+R_{SB}$	(+,*,*,*)	precarious
(1,1,1,0)	$-C_{P},B_{E}-P_{SB}-R_{SB},-P_{SN}+{NC}_{E}-R_{SN},$ $C_{S}+R_{SB}+R_{SN}$	(-,*,*,+)	precarious
(1,1,1,1)	$C_{P},C_{S},B_{E}-P_{SB}-R_{SB},$ $-P_{SN}+N_{CE}-R_{SN}$	(+, + ,*,*)	precarious

Note: * indicates undetermined positivity.

Condition 1: $P_{SN}-{NC}_{E}+R_{SN}\;>\;0$ Condition 1*:$P_{SN}-{NC}_{E}+R_{SN}\;<\;0$
Condition 2:$B_{E}-P_{SB}-R_{SB}\;>\;0$ Condition 2*: $B_{E}-P_{SB}-R_{SB}\;<\;0$
Condition 3:$C_{P}-R_{SPN}\;>\;0$.

**Fig 3:**
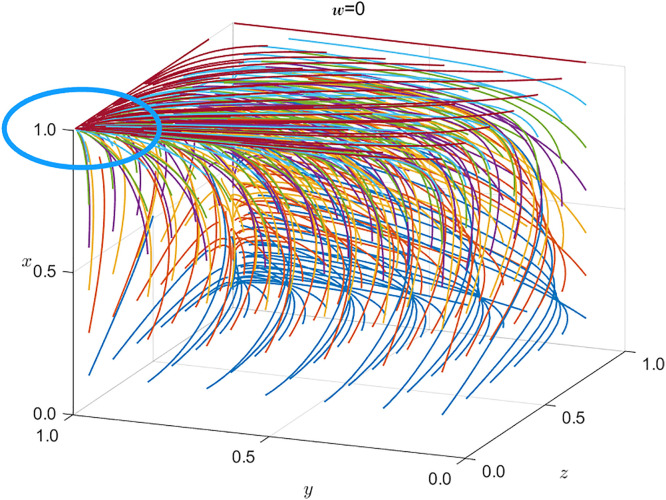
Evolution of tripartite strategies under congregational negative surveillance (see Table 8 for parameter values).

Under conditions 1, 2, and 3, SRSA’s benefits from strict regulation exceed those from non-regulation, so SRSA selects strict supervision. For China Railway Nanchang Group Co., Ltd., the expected penalties and rewards under strict SRSA oversight outweigh any savings from reduced safety investment; hence, bounded-rational profit maximization induces safe operations. In contrast, local safety-critical enterprises find that the same savings exceed the expected penalties and rewards, so they rationally adopt unsafe practices. Finally, the public’s expected reward for active supervision is lower than the associated cost, leading the public not to supervise. The resulting strategic dynamics is illustrated in Fig 3.

**Fig 4:**
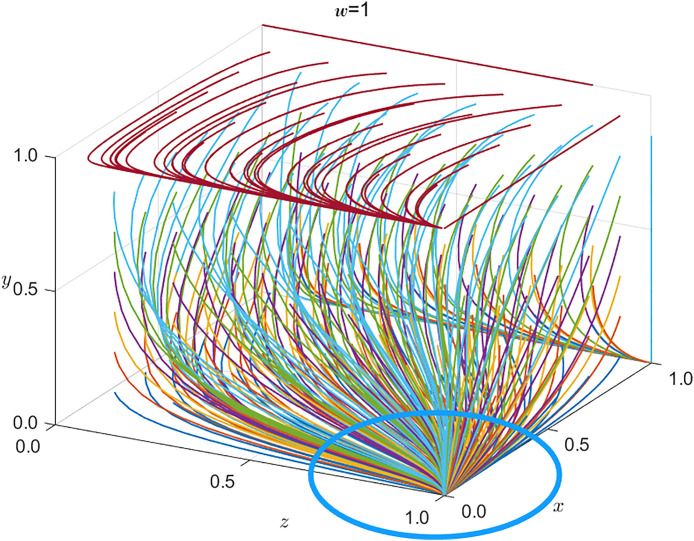
Evolution of tripartite strategies under active supervision (see Table 8 for parameters).

Under conditions 1* and 2*, which are the opposite of conditions 1 and 2, the SRSA’s payoff from strict regulation falls below that from non-regulation; nevertheless, SRSA still selects strict supervision. For China Railway Nanchang Group Co., Ltd., the savings from reduced safety investment exceed the combined rewards and penalties under strict oversight, yet the firm opts for safe operations. Local safety-critical enterprises likewise find the same savings outweighed by the expected sanctions and incentives, so they rationally choose safe practices. Finally, the public’s expected reward for active supervision surpasses the associated cost, inducing the public to supervise. The resulting strategic dynamics is illustrated in Fig 4.

### System dynamics simulation

#### Model construction and initialization.

According to the analysis of the above evolutionary game theory in Section 3.2, it can be seen that a fixed value of reward and punishment cannot achieve the minimization of the supervisory input of SRSA, and the maximization of the safety productivity of the China Railway Nanchang Bureau Group Co., Ltd., the local safety auxiliary enterprises, and the public supervision rate. Therefore, we implement a system dynamics model of the four-party evolutionary game (SRSA, China Railway Nanchang Bureau Group Co., Ltd., safety-supportive enterprises, public) to stabilize strategic evolution. This approach visually simulates dynamic adaptation processes in railway safety supervision and quantifies inter-strategy influence relationships.

**Fig 5:**
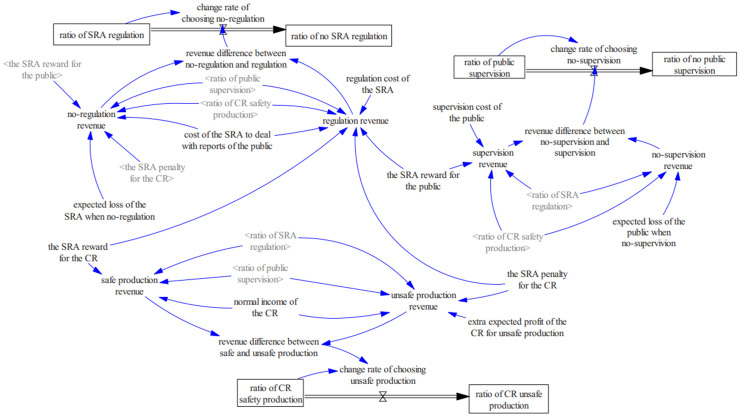
The diagram of the system dynamics model.

A system dynamics model is developed using Vensim PLE 10.3.4, implementing evolutionary game theory through a multi-agent architecture (methodological details in S2 File Appendix C). The model integrates four interactive subsystems: SRSA regulatory agents, China Railway Nanchang Bureau Group Co., Ltd. operational entities, regional safety-critical enterprise coalitions, and public stakeholder [11]. As depicted in Fig 5, this computational construct simulates coevolutionary processes among regulators, operators, and civil society within China’s railway safety governance ecosystem.

The study simulate daily strategy revisions over a 24-month horizon with the following parameters [46]: Initial time = 0; Final time = 24; Time step = 0.002; Time unit: days. The external variables shown in Table 9 are calibrated from 2023 State Railway Administration Financial Statement, 2023 China Railway Corporation Audit Report, Railway Safety Management Regulations, and an expert survey. The average daily cost of SRSA is approximately 350,000 RMB. According to the official data, the total profit of China Railway Nanchang Bureau Group Co., Ltd. is about RMB 23.8 billion. The average daily revenue of China Railway Nanchang Bureau Group Co., Ltd. is about RMB 65.2 million. Penalties for non-compliance are set at 40% of profit and safety incentives at 20% of profit according to 2022 China Railway Yearbook. The values of other variables are also taken from 2022 China Railway Yearbook. Expected losses for SRSA are government performance loss and reputation loss in the absence of regulation. The data for both components are taken from 2022 China Railway Yearbook and a survey of railroad industry experts. The public economic losses caused by railroad transportation safety accidents, as well as the values of the remaining parameters, are referenced in 2022 China Railway Yearbook.

**Table 9:** Initial values of external variables.

Norm	Significance of the indicators	Initial value (in millions)
*C*_*S*_	Supervision of Nanchang Bureau and business inputs	0.35
*C*_*SP*_	Handling of public complaint inputs	0.64
*L*_*SN*_	Losses due to inadequate supervision of Nanchang Bureau	13.7
*L*_*SB*_	Losses from inadequate supervision of local businesses	6.75
*P*_*SN*_	Fines for poor safety operations at Nanchang Bureau	26.08
*P*_*SB*_	Fines for poor safety at local businesses	13.752
*R*_*SN*_	Strictly safe operation incentives for Nanchang Bureau	13.04
*R*_*SB*_	Strict safety incentives for local businesses	6.876
*R*_*SPN*_	Rewards for public scrutiny of Nanchang Bureau	0.83
*R*_*SPB*_	Rewards for public scrutiny of local businesses	0.35
*NC*_*N*_	Revenue from day-to-day implementation of security operations	65.2
*NC*_*E*_	Operating additional revenue against security	26.08
*L*_*BN*_	Unsafe production by local businesses takes its toll	16.7
*B*_*N*_	Daily implementation of security production revenue	34.38
*B*_*E*_	Additional income from breach of security	13.752
*C*_*P*_	Supervisory expenditures on Nanchang Bureau and local enterprises	0.64
*L*_*NP*_	Neglect of unsafe operating losses at Nanchang Bureau	3.45
*L*_*BP*_	Neglect of unsafe production losses in local businesses	1.63

### Evolution of a four-party strategy with initial parameter values kept static

**Fig 6:**
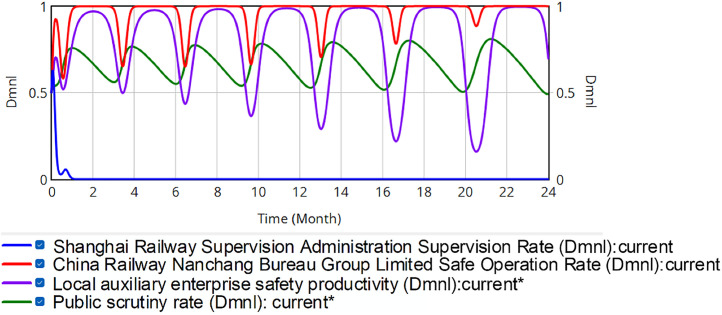
Evolution of the quadrilateral strategy.

The initial strategy selection rates of each party are set as *x* = 0.5, *y* = 0.5, *z* = 0.5, *w* = 0.5. This means that in the initial conditions, all participants have the same probability of choosing a positive or negative strategy. This situation is more common in real life. The left *y*-axis represents the initial value, and the right *y*-axis represents the range of actual fluctuations. As shown in Fig 6, the simulation results do not reach the evolutionary stable strategy.

The fluctuations of the red curve are gradually decreasing; however, fluctuations persist, while the purple and green curves exhibit sharper fluctuations. By month 17, only 22.7% of local safety-critical enterprises are operating safely, exposing Nanchang Bureau to elevated accident risk. Overall, the regulatory regime fails to reach an ESS. The strategies of China Railway Nanchang Bureau Group Co., Ltd., local safety auxiliary enterprises, and the public remain volatile, and the safety share among these enterprises is trending downward. Greater fluctuation severity correlates with higher risk in the railroad transportation safety supervision system. Analysis of the red, purple and green curve fluctuations reveals that the safety production proportion at China Railway Nanchang Bureau Group Co., Ltd. exhibits a downward trend, whereas public supervision shows an upward trend. An alternating influence relationship exists among China Railway Nanchang Bureau Group Co., Ltd., local safety-supportive enterprises, and the public. The safety productivity fluctuation cycle of China Railway Nanchang Bureau Group Co., Ltd. and local safety auxiliary enterprises lags behind the supervision rate of the public. This phenomenon occurs because when their safety productivity declines, the public increases the regulatory rate, compelling improvements. Conversely, when productivity rises sufficiently, public oversight relaxes, leading enterprises to reduce safety investments for greater profit. Consequently, cyclical fluctuations emerge between safety productivity and public supervision intensity. This indicates that public supervision partially regulates the safety productivity of China Railway Nanchang Bureau Group Co., Ltd. and local safety-supportive enterprises. In this process, SRSA encourages the public assistance in regulating these entities rather than direct intervention. In reality, this approach can alleviate the lack of human resources and reduce labor costs for government regulators. However, it fails to eliminate systemic risks. The probability of safety accidents is higher at the troughs of the red and purple curves. Regulatory uncertainty further complicates control, while local enterprises’ safety productivity declines progressively, with each cycle reaching lower minima.

**Fig 7:**
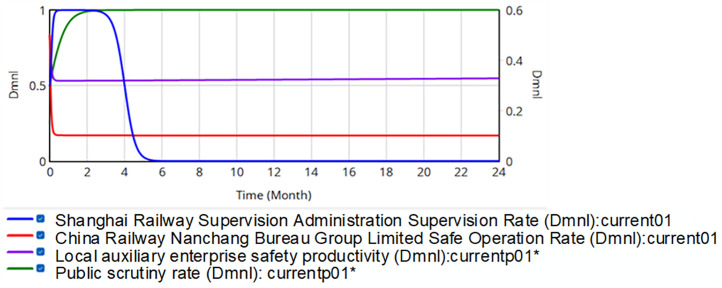
Evolution of quadratic strategy when the penalty value is 20% of the value of the safety production gain.

**Fig 8:**
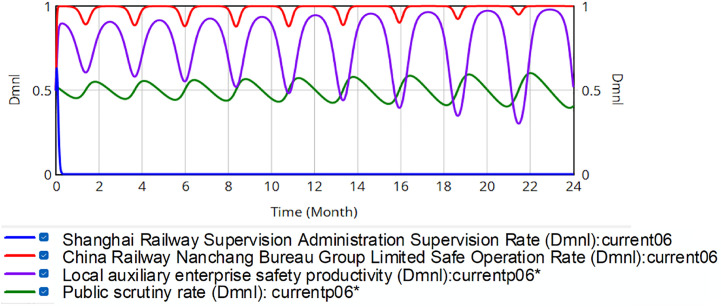
Evolution of quadratic strategy when the penalty value is 60% of the value of the safety production gain.

**Fig 9:**
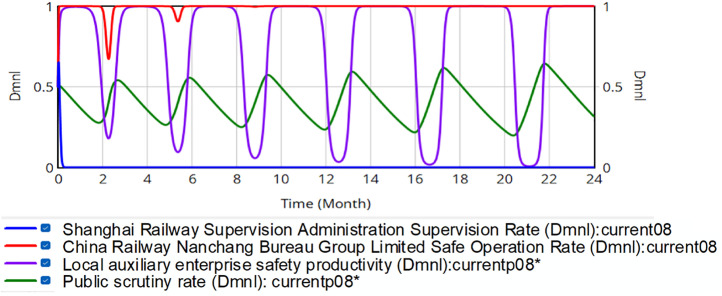
Evolution of the quadratic strategy when the penalty value is 80% of the value of the safety production gain.

In practice, increasing fines achieves regulatory equilibrium. Holding other factors constant, reward-penalty ratios should be adjusted. The study test this via systematic sensitivity analysis:

Scenario 1: Changing the penalty rate. Penalty intensities for China Railway Nanchang Bureau Group Co., Ltd. and regional safety-supporting enterprises are parametrically modulated at three discrete levels (20%, 60%, 80%), deviating from the baseline 40% sanction intensity. Corresponding penalties are: *P*_*SB*_ = RMB 6.876, 20.628 or 27.504 million, and *P*_*SN*_ = RMB 13.04, 39.12 or 52.16 million. Holding initial policy parameters constant as *x* = 0.5, *y* = 0.5, *z* = 0.5, *w* = 0.5, Figs 7–9 present the simulation results for penalty coefficients of 20%, 60% and 80%, respectively. The left *y*-axis represents the initial value, and the right *y*-axis represents the range of actual fluctuations.

Comparison of Figs 6 and 7 demonstrates that the system reaches equilibrium after SRSA reduces penalties to 20% of the safety production revenue of the China Railway Nanchang Bureau Group Co., Ltd. This indicates that detected unsafe production practices would not impact the corporation’s overall profitability. Extremely low penalty rates could trigger systemic regulatory failure.

Comparison of Figs 6 and 8 indicates that after SRSA increased the percentage of fines to 60% of the safety production revenue of China Railway Nanchang Bureau Group Co., Ltd., the downward fluctuation in its safety production rate decelerates. However, systemic risks persist while auxiliary enterprises exhibit heightened volatility in safety production. These results indicate that although 60% penalty rates mitigate fluctuation risks, they fail to eliminate the risk.

Comparison of Figs 6 and 9 indicates that when SRSA imposes an 80% penalty rate on the safety production revenue of China Railway Nanchang Bureau Group Co., Ltd., the red curve gradually leveled off. In contrast, the purple curve fluctuates more seriously. The evolutionary results show that a higher fine rate increases the risk of volatility in the railroad transportation safety supervision system.

**Fig 10:**
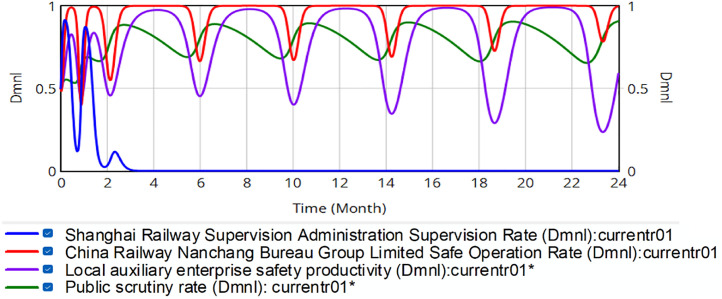
Evolution of quadrilateral strategies for a reward value of 10% of safety production gain.

**Fig 11:**
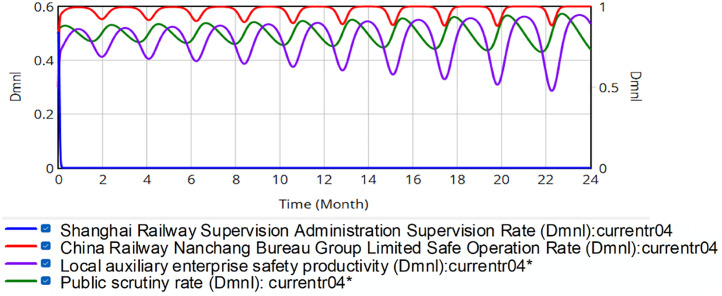
Evolution of quadrilateral strategies for a reward value of 20% of the value of safety production gain.

Scenario 2: Changing the incentive rate. The study modulates incentive rates *R*_*SN*_ and *R*_*SB*_ from 20% to 10% or 40% for the China Railway Nanchang Bureau Group Co., Ltd. and local security support enterprises, i.e., *R*_*SN*_ = RMB 6.52 or RMB 26.08 million and *R*_*SB*_ = RMB 13.752 or RMB 3.438 million. Holding initial policy parameters constant as *x* = 0.5, *y* = 0.5, *z* = 0.5, *w* = 0.5, Figs 10 and 11 present simulation outcomes for 10% and 40% incentive coefficients, respectively. The left *y*-axis represents the initial value, and the right *y*-axis represents the range of actual fluctuations.

Comparison of Figs 6 and 10 shows that reducing SRSA incentives to 10% of safety production revenue for China Railway Nanchang Bureau Group Co., Ltd. and safety-supportive enterprises amplifies volatility in their safety production metrics. The persistent systemic risk demonstrates that insufficient incentives exacerbate regulatory instability.

Comparing Figs 6 with 11, it can be seen that 40% incentive rates induce stabilization in both safety indicators of the China Railway Nanchang Bureau Group Co., Ltd. (red curve) and auxiliary enterprises’ performance (see purple curve). These evolutionary patterns confirm that moderately increased incentives effectively mitigate fluctuation risks within China’s rail transportation safety framework.

### Evolution of four-party strategy under dynamic change of initial parameters

Extensive research confirms that linking penalty severity to unsafe behavior rates effectively mitigates system volatility. This study applies an adaptive penalty framework to China’s rail safety oversight system, establishing proportionality between enforcement intensity and quantified risk exposure metrics. Then we denote the dynamic penalty by *P*_SN_^*^ and *P*_SB_^*^, and the equations are as follows:


$$P_{SN}^{*}=\;(1-y)\;P_{SN}$$ (15)

$$P_{SB}^{*}=\;(1-z)\;P_{SB}$$ (16)

Replace the parameters *P*_*SN*_ and *P*_*SB*_ with Eqs (15) and (16).

**Fig 12:**
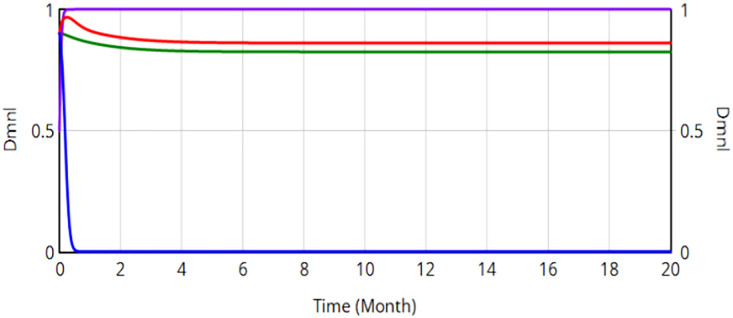
Evolution of quadratic strategy when penalty value is dynamically adjusted.

A dynamic punishment mechanism is introduced into the system dynamics model of the evolutionary game of railroad transportation safety regulation in China. To simulate diverse real-world scenarios while maintaining parametric consistency, four strategy coefficients represent the initial strategic orientations (neutral, negative, or positive) of SRSA, the China Railway Nanchang Bureau Group Co., Ltd., local safety auxiliary enterprises and the public, respectively. The evolution results are presented in Fig 12.

According to Fig 12, all four curves eventually reach a steady state, i.e., no participant changes the strategy. The safety production rate of the China Railway Nanchang Bureau Group Co., Ltd. is 86.3%, the supervision rate by the SRSA is 0, and the public supervision rate is 79.2%. This configuration enables SRSA to optimize resource allocation by leveraging social oversight, effectively resolving regulatory resource dilemmas. The dynamic punishment mechanism achieves system stability by suppressing volatility, thereby promoting evolutionary convergence toward higher safety levels. However, the safety production ratio of the China Railway Nanchang Bureau Group Co., Ltd. remains suboptimal and requires further enhancement.

Given reward-penalty interdependence in strategic decision-making, the study propose a dynamic reward-punishment mechanism to strengthen regulatory efficacy. Dynamic rewards *R*_SN_^*^ and *R*_SB_^*^ correlate directly with relative safety production performance between the China Railway Nanchang Bureau Group Co., Ltd. and its auxiliary firms, formally defined as:


$$R_{SN}^{*}=\;yR_{SN}$$ (17)

$$R_{SB}^{*}=\;zR_{SB}$$ (18)

Based on dynamic rewards, the study implement parametric reconfiguration by substituting the coefficients *R*_*SN*_ and *R*_*SB*_ with time-dependent control operators formalized in Eqs (17) and (18).

**Fig 13:**
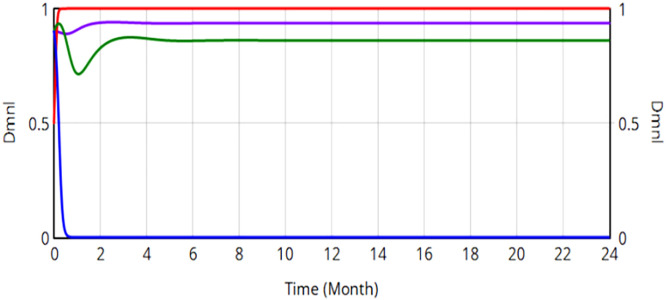
Evolution of quadrilateral strategy when penalty value reward value are dynamically adjusted.

Then a dynamic reward-punishment mechanism is incorporated into the system dynamics model of the evolutionary game of railroad transportation safety supervision. Maintaining parametric consistency, four change proportions represent the neutral, negative and positive strategies of SRSA, the China Railway Nanchang Bureau Group Co., Ltd., local safety auxiliary enterprises and the public in the initial state of the system, respectively. The evolution results are shown in Fig 13.

All stakeholder strategies rapidly converge to an ESS within the rail safety governance framework, stabilizing at (0, 0.997, 0.932, 0.872). Dynamic equation analysis confirms this equilibrium constitutes a stable ESS. The safe production percentage in the China Railway Nanchang Bureau Group Co., Ltd. is 99.7%. Post-implementation analysis reveals significant system improvements, including a 13.4% increase in industrial safety indices (Nanchang Bureau) and an 87.2% increase in societal monitoring engagement. The dynamic reward-punishment mechanism can effectively suppress volatility, reducing systemic risk while enhancing stability in the safety performance of China Railway Nanchang Bureau Group Co., Ltd. Crucially, the dynamic reward-punishment mechanism outperforms penalty-only frameworks by further optimizing safety production ratios.

These results demonstrate that integrating public supervision with a dynamic reward-punishment mechanism strengthens rail transportation safety oversight. This approach enables governments to reduce direct monitoring costs by leveraging participatory governance. As mentioned earlier, the regulatory function has undergone changes following the reform of China’s railroad institutions. The independence of the regulatory body has increased, and the regulatory relationship has shifted from internal to external. The regulatory system is reorganized from a four-level hierarchical structure to a two-level hierarchical structure. The number of regulatory practitioners has been significantly reduced. Under these constraints, SRSA’s limited inspection capacity (evidenced by published coverage statistics) renders real-time supervision of the China Railway Nanchang Bureau Group Co., Ltd. infeasible. Due to the large number of trains running and train stations every day, SRSA can only conduct a small number of inspections. Therefore, in reality, the proportion of safety supervision by SRSA is not high. The model simulation results are consistent with this situation. Based on this, the results of this study indicate that public participation in the regulatory system can effectively alleviate this problem. The public serves as both a purchaser and a regulator of the services provided by China Railway Nanchang Bureau Group Co., Ltd. The public can provide SRSA with the information about the unsafe production of the China Railway Nanchang Bureau Group Co., Ltd. and the local safety-supporting enterprises. The cost of using these information for regulatory purposes is lower than the cost of direct regulation.

## Conclusions

This study establishes a comprehensive safety management system for China’s railway transportation systems through multi-stakeholder analysis, integrating social forces and auxiliary enterprises within an evolutionary game model. The study conducts system dynamics simulations to quantify public participation effects on the safety production of the China Railway Nanchang Bureau Group Co., Ltd., analyzes reward-punishment mechanism efficacy on strategic evolution, and develops dynamic reward-punishment mechanism suppressing regulatory volatility.

This work represents a breakthrough beyond conventional static regulatory paradigms by constructing dynamic correlations between time-variant incentive functions and the evolution of collective strategies. Results demonstrate that the introduced dynamic reward-punishment mechanism is effective in dampening fluctuations in the regulatory evolution process and reducing safety risks. Notably, the study demonstrates a mutually reinforcing relationship between public oversight intensity and corporate safety investments, validating the applicability of social co-governance models in railway safety management. Given the current lack of empirical verification in the research, the simulation results will be further validated through experiments in the future. In addition, some studies will be conducted to examine some of the assumptions in the paper, such as dynamically analyzing the effects of participants with varying levels of knowledge of safety regulations, which will further enrich the comprehensiveness of the research.

## Supporting information

S1 File Procedure codes of payoff matrix calculation. (DOCX)

S2 File Appendices. Appendix A-Expressions of Expected Return. Appendix B-Jacobi Matrix of the Four-party Evolutionary Game System. Appendix C-Vensim Simulation Platform.
(DOCX)

S3 File Procedure codes of Jacobi matrix solution. (DOCX)
