# Supplementary material for: Modeling and simulation of railway safety management with public supervision and dynamic incentives: A four-party evolutionary game and system dynamics approach
Source: PLoS One. 2025 Aug 18;20(8):e0330100. doi: 10.1371/journal.pone.0330100 (PMC12360609; doi:10.1371/journal.pone.0330100)
Supplement: S1 File — (DOCX) [file pone.0330100.s001.docx]

**Procedure codes of payoff matrix calculation**

syms x y z w Cs Csp Lsn Lsb Psn Psb Rsn Rsb NCn NCe Bn Be Lbn Cp Lpn Lpb Rspn Rspb;

Cs=4.66;Csp=0.41;Lsn=13.7;Lsb=13.7;Psn=65.75;Psb=65.75;Rsn=32.87;Rsb=32.87;NCn=164.38;NCe=65.75;Bn=164.38;Be=65.75;Lbn=34.25;Cp=0.82;Lpn=2.74;Lpb=2.74;Rspn=2.74;Rspb=2.74;

S1=y*z*w*(-Cs-Csp-Rsn-Rsb)+y*z*(1-w)*(-Cs-Rsn-Rsb)+y*(1-z)*w*(-Cs-Csp+Psb-Rsn-Rspb)+y*(1-z)*(1-w)*(-Cs+Psb-Rsn)+(1-y)*z*w*(-Cs-Csp+Psn-Rsb-Rspn)+(1-y)*z*(1-w)*(-Cs+Psn-Rsb)+(1-y)*(1-z)*w*(-Cs-Csp+Psn+Psb-Rspn-Rspb)+(1-y)*(1-z)*(1-w)*(-Cs+Psn+Psb);

S2=y*z*w*(-Csp-Rsn-Rsb)+y*z*(1-w)*0+y*(1-z)*w*(-Csp+Psb-Rsn-Rspb)+y*(1-z)*(1-w)*(-Lsb)+(1-y)*z*w*(-Csp+Psn-Rsb-Rspn)+(1-y)*z*(1-w)*(-Lsn)+(1-y)*(1-z)*w*(-Csp+Psn+Psb-Rspb-Rspn)+(1-y)*(1-z)*(1-w)*(-Lsn-Lsb);

S=simplify(x*S1+(1-x)*S2);

dfdx=simplify(x*(S1-S));

N1=x*z*w*(NCn+Rsn)+x*z*(1-w)*(NCn+Rsn)+x*(1-z)*w*(NCn+Rsn)+x*(1-z)*(1-w)*(NCn+Rsn)+(1-x)*z*w*(NCn+Rsn)+(1-x)*z*(1-w)*(NCn)+(1-x)*(1-z)*w*(NCn+Rsn)+(1-x)*(1-z)*(1-w)*(NCn-Lbn);

N2=x*z*w*(NCn+NCe-Psn)+x*z*(1-w)*(NCn+NCe-Psn)+x*(1-z)*w*(NCn+NCe-Psn)+x*(1-z)*(1-w)*(NCn+NCe-Psn)+(1-x)*z*w*(NCn+NCe-Psn)+(1-x)*z*(1-w)*(NCn+NCe)+(1-x)*(1-z)*w*(NCn+NCe-Psn)+(1-x)*(1-z)*(1-w)*(NCn+NCe-Lbn);

N=simplify(y*N1+(1-y)*N2);

dfdy=simplify(y*(N1-N));

B1=x*y*w*(Bn+Rsb)+x*y*(1-w)*(Bn+Rsb)+x*(1-y)*w*(Bn+Rsb)+x*(1-y)*(1-w)*(Bn+Rsb)+(1-x)*y*w*(Bn+Rsb)+(1-x)*y*(1-w)*(Bn)+(1-x)*(1-y)*w*(Bn+Rsb)+(1-x)*(1-y)*(1-w)*(Bn);

B2=x*y*w*(Bn+Be-Psb)+x*y*(1-w)*(Bn+Be-Psb)+x*(1-y)*w*(Bn+Be-Psb)+x*(1-y)*(1-w)*(Bn+Be-Psb)+(1-x)*y*w*(Bn+Be-Psb)+(1-x)*y*(1-w)*(Bn+Be)+(1-x)*(1-y)*w*(Bn+Be-Psb)+(1-x)*(1-y)*(1-w)*(Bn+Be);

B=simplify(z*B1+(1-z)*B2);

dfdz=simplify(z*(B1-B));

P1=x*y*z*(-Cp)+x*y*(1-z)*(-Cp+Rspb)+x*(1-y)*z*(-Cp+Rspn)+x*(1-y)*(1-z)*(-Cp+Rspn+Rspb)+(1-x)*y*z*(-Cp)+(1-x)*y*(1-z)*(-Cp+Rspb)+(1-x)*(1-y)*z*(-Cp+Rspn)+(1-x)*(1-y)*(1-z)*(-Cp+Rspn+Rspb);

P2=x*y*z*0+x*y*(1-z)*0+x*(1-y)*z*0+x*(1-y)*(1-z)*0+(1-x)*y*z*0+(1-x)*y*(1-z)*(-Lpb)+(1-x)*(1-y)*z*(-Lpn)+(1-x)*(1-y)*(1-z)*(-Lpn-Lpb);

P=simplify(w*P1+(1-w)*P2);

dfdw=simplify(w*(P1-P)).
