# Supplementary material for: Modeling and simulation of railway safety management with public supervision and dynamic incentives: A four-party evolutionary game and system dynamics approach
Source: PLoS One. 2025 Aug 18;20(8):e0330100. doi: 10.1371/journal.pone.0330100 (PMC12360609; doi:10.1371/journal.pone.0330100)
Supplement: S2 File — Appendix A-Expressions of Expected Return. Appendix B-Jacobi Matrix of the Four-party Evolutionary Game System. Appendix C-Vensim Simulation Platform. (DOCX) [file pone.0330100.s002.docx]

**Appendix A**

**Expressions of Expected Return**

*S*_1_*=yzw*(*-C_S_-C_SP_-R_SN_-R_SB_*)*+yz*(1*-w*)(*-C_S_-R_SN_-R_SB_*)*+y*(1*-z*)

*w*(*-C_S_-C_SP_+P_SB_-R_SN_-R_SPB_*)*+y*(1*-z*)(1*-w*)(*-C_S_+P_SB_-R_SN_*)

*+*(1*-y*)*zw*(*-C_S_-C_SP_+P_SN_-R_SB_-R_SPN_*)+(1*-y*)*z*(1*-w*) (1)

(-*C_S_+P_SN_-R_SB_*)*+*(1*-y*)(1*-z*)*w*(*-C_S_-C_SP_+P_SB_+P_SN_*

*-R_SPN_-R_SPB_*)+(1*-y*)(1*-z*)(1*-w*)(*-C_S_+P_SB_+P_SN_*)

*S*_2_*=yzw*(*-C_SP_-R_SN_-R_SB_*)*+y*(1*-z*)*w*(*-C_SP_+P_SB_-R_SN_-R_SPB_*)*+y*(1*-z*)

(1*-w*)(-*L_SB_*)+(1*-y*)*zw*(*-C_SP_+P_SN_-R_SB_-R_SPN_*)+(1*-y*)*z*(1*-w*)

(*-L_SN_*)+(1*-y*)(1*-z*)*w*(*-C_SP_+P_SB_+P_SN_-R_SPN_-R_SPB_*) (2)

*+*(1*-y*)(1*-z*)(1*-w*)(*- L_SN_-L_SB_*)

*NC*_1_*=xzw*(*NC_N_+R_SN_*)*+xz*(1*-w*)(*NC_N_+R_SN_*)*+x*(1*-z*)*w*(*NC_N_+R_SN_*)*+x*

(1*-z*)(1*-w*)(*NC_N_+R_SN_*)+(1*-x*)*zw*(*NC_N_+R_SN_*)+(1*-x*)*z*(1*-w*)(*NC_N_*) (3)

*+*(1*-x*)(1*-z*)*w*(*NC_N_+R_SN_*)+(1*-x*)(1*-z*)(1*-w*)(*NC_N_-L_BN_*)

*NC*_2_*=xzw*(*NC_N_+NC_E_-P_SN_*)*+xz*(1*-w*)(*NC_N_+NC_E_-P_SN_*)*+x*(1*-z*)*w*

(*NC_N_+NC_E_-P_SN_*)*+x*(1*-z*)(1*-w*)(*NC_N_+NC_E_-P_SN_*)+(1*-x*)*zw*

(*NC_N_+NC_E_-P_SN_*)+(1*-x*)*z*(1*-w*)(*NC_N_+NC_E_*)+(1*-x*)(1*-z*) (4)

*w*(*NC_N_+NC_E_-P_SN_*)+(1*-x*)(1*-z*)(1*-w*)(*NC_N_+NC_E_-L_BN_*)

*B*_1_*=xyw*(*B_N_+R_SB_*)*+xy*(1*-w*)(*B_N_+R_SB_*)*+x*(1*-y*)*w*(*B_N_+R_SB_*)+*x*(1-*y*)

(1*-w*)(*B_N_+R_SB_*)+(1*-x*)*yw*(*B_N_+R_SB_*)+(1*-x*)*y*(1*-w*)(*B_N_*) (5)

*+*(*1-x*)(1*-y*)*w*(*B_N_+R_SB_*)*+*(1*-x*)(1*-y*)(1*-w*)(*B_N_*)

*B*_2_*=xyw*(*B_N_+B_E_-P_SB_*)*+xy*(1*-w*)(*B_N_+B_E_-P_SB_*)+*x*(1*-y*)*w*(*B_N_+B_E_-P_SB_*)

*+x*(1*-y*)(1-*w*)(*B_N_+B_E_-P_SB_*)+(1-*x*)*yw*(*B_N_+B_E_-P_SB_*)+(1*-x*)*y*(1*-w*) (6)

(*B_N_+B_E_*)+(1*-x*)(1*-y*)*w*(*B_N_+B_E_-P_SB_*)+(1*-x*)(1*-y*)(1*-w*)(*B_N_+B_E_*)

*P*_1_*=xyz*(-*C_SP_*)*+xy*(1*-z*)(*-C_SP_+R_SPB_*)*+x*(1*-y*)*z*(*-C_SP_+R_SPN_*)*+x*(1*-y*)(1*-z*)

(*-C_SP_+R_SPN_+R_SPB_*)+(1*-x*)*yz*(*-C_SP_*)+(1*-x*)*y*(1*-z*)(*-C_SP_+R_SPB_*)+(1*-x*) (7)

(1*-y*)*z*(*-C_SP_+R_SPN_*)+(1*-x*)(1*-y*)(1*-z*)(*-C_SP_+R_SPB_+R_SPN_*)

*P*_2_*=*(1*-x*)*y*(1*-z*)(*-L_BP_*)+(1*-x*)(1*-y*)*z*(*-L_NP_*)

*+*(1*-x*)(1*-y*)(1*-z*)(*-L_NP_-L_BP_*) (8)

**Appendix B**

**Jacobi Matrix of the Four-party Evolutionary Game System**

$$\left[\begin{matrix} \frac{\partial f}{\partial x} & \frac{\partial f}{\partial y} & \frac{\partial f}{\partial z} & \frac{\partial f}{\partial w} \\ \frac{\partial g}{\partial x} & \frac{\partial g}{\partial y} & \frac{\partial g}{\partial z} & \frac{\partial g}{\partial w} \\ \frac{\partial h}{\partial x} & \frac{\partial h}{\partial y} & \frac{\partial h}{\partial z} & \frac{\partial h}{\partial w} \\ \frac{\partial m}{\partial x} & \frac{\partial m}{\partial y} & \frac{\partial m}{\partial z} & \frac{\partial m}{\partial w} \end{matrix}\right]$$

Where $f=\frac{dx}{dt}$, $g=\frac{dy}{dt}$, $h=\frac{dz}{dt}$, $m=\frac{dw}{dt}$.

**Appendix C**

**Vensim Simulation Platform**

Ventana Systems specializes in enterprise-grade computational modeling platforms that synthesize operational workflows and technological infrastructures, providing system dynamics solutions for strategic decision-making in complex organizational ecosystems. The proprietary simulation language Vensim was developed through a combination of existing products and custom code, creating a powerful system dynamics modeling tool. Vensim facilitates user cognition and communication through graphical cause-and-effect loops and flowcharts, supports the development of multiple model views, and provides an efficient environment for writing equations [1]. Capable of accommodating models from a few to millions of variables, it incorporates modern simulation architectures with multi-modal verification protocols including input validation frameworks featuring adaptive test vector configurations with boundary condition injection; logic formalization modules implementing Boolean gate operations via propositional calculus; stochastic process emulators utilizing Monte Carlo engines with configurable probability distribution kernels; temporal dynamics controllers integrating hybrid delay systems of continuous-time differential equations and discrete-event queuing models. This computational infrastructure enables robust verification of complex system behaviors through systematic integration of deterministic and stochastic operational parameters. Vensim’s simulation engine operates rapidly while supporting large datasets for model parameterization via external data inputs and simulation output validation. Additionally, Vensim provides tools for strategy optimization and sensitivity analysis to enhance model performance.

**Reference**

1. Mohammadi A, Tavakolan M, Khosravi Y. Developing safety archetypes of construction industry at project level using system dynamics. J Safety Res. 2018;67(12):17-26. doi: 10.1016/j.jsr.2018.09.010.
