# Supplementary material for: Modeling and simulation of railway safety management with public supervision and dynamic incentives: A four-party evolutionary game and system dynamics approach
Source: PLoS One. 2025 Aug 18;20(8):e0330100. doi: 10.1371/journal.pone.0330100 (PMC12360609; doi:10.1371/journal.pone.0330100)
Supplement: S3 File — (DOCX) [file pone.0330100.s003.docx]

**Procedure codes of Jacobi matrix solution**

A=[diff(dfdx,x) diff(dfdx,y) diff(dfdx,z) diff(dfdx,w);

diff(dfdy,x) diff(dfdy,y) diff(dfdy,z) diff(dfdy,w);

diff(dfdz,x) diff(dfdz,y) diff(dfdz,z) diff(dfdz,w);

diff(dfdw,x) diff(dfdw,y) diff(dfdw,z) diff(dfdw,w)];

[x*(Cs - Lsb - Lsn - Psb - Psn + Lsb*w + Lsn*w + Lsn*y + Lsb*z + Psb*w + Psn*w + Psn*y + Psb*z + Rsn*y + Rsb*z - Lsn*w*y - Lsb*w*z - Psn*w*y - Psb*w*z - Rsn*w*y - Rsb*w*z) + (x - 1)*(Cs - Lsb - Lsn - Psb - Psn + Lsb*w + Lsn*w + Lsn*y + Lsb*z + Psb*w + Psn*w + Psn*y + Psb*z + Rsn*y + Rsb*z - Lsn*w*y - Lsb*w*z - Psn*w*y - Psb*w*z - Rsn*w*y - Rsb*w*z), x*(x - 1)*(Lsn + Psn + Rsn - Lsn*w - Psn*w - Rsn*w), x*(x - 1)*(Lsb + Psb + Rsb - Lsb*w - Psb*w - Rsb*w), -x*(x - 1)*(Lsn*y - Lsn - Psb - Psn - Lsb + Lsb*z + Psn*y + Psb*z + Rsn*y + Rsb*z) -y*(y - 1)*(Psn + Rsn - Psn*w - Rsn*w), - (y - 1)*(Psn*w - NCe + Psn*x + Rsn*w + Rsn*x - Psn*w*x - Rsn*w*x) - y*(Psn*w - NCe + Psn*x + Rsn*w + Rsn*x - Psn*w*x - Rsn*w*x), -y*(y - 1)*(Psn + Rsn - Psn*x - Rsn*x) -z*(z - 1)*(Psb + Rsb - Psb*w - Rsb*w), 0, - (z - 1)*(Psb*w - Be + Psb*x + Rsb*w + Rsb*x - Psb*w*x - Rsb*w*x) - z*(Psb*w - Be + Psb*x + Rsb*w + Rsb*x - Psb*w*x - Rsb*w*x), -z*(z - 1)*(Psb + Rsb - Psb*x - Rsb*x) w*(w - 1)*(Lpb + Lpn - Lpn*y - Lpb*z), w*(w - 1)*(Lpn + Rspn - Lpn*x), w*(w - 1)*(Lpb + Rspb - Lpb*x), w*(Cp - Lpb - Lpn - Rspb - Rspn + Lpb*x + Lpn*x + Lpn*y + Lpb*z + Rspn*y + Rspb*z - Lpn*x*y - Lpb*x*z) + (w - 1)*(Cp - Lpb - Lpn - Rspb - Rspn + Lpb*x + Lpn*x + Lpn*y + Lpb*z + Rspn*y + Rspb*z - Lpn*x*y - Lpb*x*z)

equ1=x*(x - 1)*(Cs - Lsb - Lsn - Psb - Psn + Lsb*w + Lsn*w + Lsn*y + Lsb*z + Psb*w + Psn*w + Psn*y + Psb*z + Rsn*y + Rsb*z - Lsn*w*y - Lsb*w*z - Psn*w*y - Psb*w*z - Rsn*w*y - Rsb*w*z);

equ2=-y*(y - 1)*(Psn*w - NCe + Psn*x + Rsn*w + Rsn*x - Psn*w*x - Rsn*w*x);

equ3=-z*(z - 1)*(Psb*w - Be + Psb*x + Rsb*w + Rsb*x - Psb*w*x - Rsb*w*x);

equ4=w*(w - 1)*(Cp - Lpb - Lpn - Rspb - Rspn + Lpb*x + Lpn*x + Lpn*y + Lpb*z + Rspn*y + Rspb*z - Lpn*x*y - Lpb*x*z);

[a,b,c,d]=solve(equ1,equ2,equ3,equ4,x,y,z,w).
